# All in this together? Isolation and housing in ‘lockdown London’

**DOI:** 10.1111/1469-8676.12874

**Published:** 2020-05-19

**Authors:** Constance Smith

**Affiliations:** ^1^ Social Anthropology University of Manchester Manchester M13 9PL UK

‘Now we really are all in this together’. How many times have I heard this over the last few weeks? It’s a corona‐time sentiment that seems to have spanned the political spectrum,^1^ in the UK at least. It references the refrain of David Cameron’s government in post‐financial crisis Britain; it was the surface justification for the austerity politics that shaped the 2010s. When banks were ‘too big to fail’ but the welfare state was stripped to a punitive minimum, it didn’t take much to point out the insincerity of his claim.

As COVID‐19 spreads across the globe, it is tempting to suggest that maybe, this time, we really are all in it together. Almost everyone is affected in some way, but to suggest some kind of commonality of experience is to mask huge inequalities. Experiences of lockdown are drastically altered by housing and space. While the rich can escape to second homes,^2^ cramped housing and lack of outdoor space make extended time at home much harder to endure.

The very capacity to live ‘in lockdown’ is itself a privilege. Since January 2020, I have been doing fieldwork with a North London housing drop‐in centre. Led by local volunteers, it was founded in response to austerity politics to build solidarity and community in a neighbourhood with high levels of homelessness and housing precarity. Since mid‐March, the centre has transitioned to a ‘socially distanced’ service, primarily through phone‐based support. This includes connecting rough sleepers with a London‐wide strategy to use empty hotels as temporary accommodation. It makes sense to get people off the streets, but councils have relocated people all over London with little other support. To alleviate this, the centre distributes mobile phones and has established new cross‐city connections to provide food and other essentials. But, lonely and frustrated, some are refusing hotel rooms in favour of continued rough sleeping.

Visible here is a new politics of isolation, not togetherness. In times of coronavirus, breaking (physical) social ties is now presented as a goal. For community projects designed to overcome isolation, to create a different ‘assemblage of care’ (Lancione 2014) to that provided by the state, this is fundamentally disorienting. For rough sleepers already marginal from society, it can feel as though they are being locked up in these hotels, once again swept out of sight. They do not just need shelter: their social networks and knowledge of a particular terrain are vital, enabling access to anything from hot meals to companionship. A hotel room on the other side of London might help stem coronavirus infection, but it breaks already‐precarious networks of care. As one man said, ‘I want to feel safe, I want a door, but I need my people’.

Other centre users have a roof over their heads but, housed in poor‐quality, temporary accommodation, their homes feel less a refuge from coronavirus than a materialisation of its uncertainty. One family of four are living day‐to‐day, the husband laid off from a zero‐hours contract job he had just started two weeks previously. The parents are in debt, with electric that will last a week and £4 in their pockets. They are stressed, do not speak much English and don’t have a community network. The centre is providing emotional and practical support, but for families like these, lockdown amplifies the precarity and isolation of home life.

Are we really all in this together? The expenditure by Boris Johnson’s government may alleviate the immediate crisis for some. But this is not just about the impact of a novel virus, but about how it compounds the slow violence wrought by the last decade of austerity and longer‐term disinvestment in the social and physical fabric of London neighbourhoods (Watt and Minton 2016). In this community that has suffered years of cuts and neglect, horizons of coping are day‐to‐day and togetherness feels a long way off.

## Footnotes

^1^
See https://www.theguardian.com/commentisfree/2020/mar/29/coronavirus‐means‐we‐really‐are‐finally‐all‐in‐this‐together#comment‐139332930 (Accessed May 2020).

^2^
See https://www.france24.com/en/20200406‐tale‐of‐two‐cities‐how‐virus‐lockdown‐has‐exposed‐france‐s‐class‐divide (Accessed May 2020).
